# Pairwise Respiratory Viral Co-Detection Patterns Before, During, and After the COVID-19 Pandemic: An 18-Year Multiplex PCR Surveillance Study

**DOI:** 10.3390/microorganisms14051134

**Published:** 2026-05-16

**Authors:** Bo Kyeung Jung, Jeong Su Han, Jae Kyung Kim

**Affiliations:** 1 Department of Laboratory Medicine, College of Medicine, Dankook University, Cheonan-si 31116, Republic of Korea; lovegodmother@hanmail.net; 2 Department of Biomedical Laboratory Science, College of Health Sciences, Dankook University, Cheonan-si 31116, Republic of Korea; jshan1162@naver.com

**Keywords:** COVID-19 pandemic, molecular epidemiology, multiplex PCR, pairwise co-detection, respiratory virus, respiratory virus surveillance, rhinovirus, viral co-detection

## Abstract

The coronavirus disease pandemic impacted respiratory virus circulation and seasonality, but its effect on viral co-detection patterns remains unclear. We examined temporal changes in co-detection burden and dominant pairwise patterns across pandemic periods. We analyzed 23,284 respiratory virus multiplex PCR tests performed at a tertiary care center in the Republic of Korea from 2007 to 2024. Co-detection was defined as detection of ≥2 viruses in a single episode. To address temporal panel changes, we used crude full-panel, restricted 12-target, and recent-period sensitivity analyses based on a stable respiratory virus target set. Co-detection burden showed discordant trends across analytical approaches. In the crude analysis, co-detection among positive episodes was the highest during the pandemic. In the restricted analysis, co-detection decreased from 20.5% before to 14.8% during and 12.2% after the pandemic. Pairwise co-detection patterns also shifted: adenovirus–rhinovirus predominated before the pandemic, whereas rhinovirus-containing pairs involving parainfluenza virus type 3 or respiratory syncytial virus B accounted for relatively larger shares during and, to a lesser extent, after the pandemic. These findings suggest that post-pandemic respiratory virus surveillance should consider not only single-virus positivity or overall co-detection frequency, but also the composition of dominant pairwise viral combinations captured by multiplex PCR.

## 1. Introduction

Respiratory viruses are major causes of acute respiratory illness across all age groups and serve as key seasonal drivers of outpatient visits, emergency department utilization, and hospitalization [1]. The widespread adoption of multiplex PCR has enabled the simultaneous and high-sensitivity detection of multiple viral targets, thereby expanding respiratory virus surveillance beyond single-pathogen positivity rates to include patterns of viral co-detection within individual specimens [2]. This shift has highlighted the need to interpret respiratory virus epidemiology not only at the level of individual pathogens but also in the context of co-circulation and co-detection dynamics [3].

The coronavirus disease (COVID-19) pandemic substantially altered the circulation of respiratory viruses beyond severe acute respiratory syndrome coronavirus 2 [4]. Non-pharmaceutical interventions, including mask-wearing, social distancing, school closures, and changes in healthcare-seeking behavior, were associated with marked reductions in the detection of seasonal respiratory viruses, such as influenza virus, respiratory syncytial virus (RSV), and parainfluenza virus (PIV), followed by heterogeneous recovery patterns across different viruses [5,6]. These observations suggest that the pandemic influenced not only the magnitude of viral epidemics but also the broader ecological landscape in which multiple respiratory viruses co-circulate [7].

Even after the relaxation of control measures, respiratory viruses did not revert to pre-pandemic patterns. Asynchronous or atypical resurgences have been reported for several viruses, particularly RSV and PIV, whereas rhinovirus has shown relatively persistent or earlier re-emergence compared with some enveloped viruses [8]. These findings indicate that post-pandemic changes in respiratory virus epidemiology cannot be fully explained by alterations in individual virus positivity or seasonality alone and that temporal overlap and co-detection structures among viruses should be considered.

However, most post-pandemic surveillance studies have primarily focused on changes in detection rates, timing of epidemics, or age-specific distributions of individual viruses [9]. In contrast, relatively few studies have examined how the composition of viral co-detection and dominant pairwise structures have been reorganized across the pre-pandemic, pandemic, and post-pandemic periods. This question is epidemiologically relevant because co-detection patterns may reflect temporal overlap among viruses, shifts in the tested populations, and changes in viral communities captured by multiplex assays [10]. However, the detection of multiple viral nucleic acids within a single specimen does not necessarily imply synergistic pathogenic interactions; thus, careful interpretation is required.

In addition, long-term analyses using multiplex PCR data must account for methodological heterogeneity, particularly the changes in assay panel composition over time. Differences in the included targets and analytical performance across multiplex platforms may influence the temporal comparisons of the co-detection burden and composition [8,9,10]. Therefore, both crude analyses using full panels and restricted analyses based on common targets, along with sensitivity analyses, are necessary to ensure a robust interpretation of long-term surveillance data.

In this context, we used long-term multiplex PCR data from a tertiary care center in the Republic of Korea to characterize temporal changes in respiratory viral co-detection across all age groups, with particular emphasis on pairwise composition before, during, and after the COVID-19 pandemic. Specifically, we aimed to determine whether the pandemic was associated not only with changes in the overall co-detection burden, but also with redistribution of dominant pairwise viral combinations across epidemiologic periods. Furthermore, to account for potential biases related to changes in assay target composition, we performed both restricted analyses using common targets and a recent-period sensitivity analysis using data from 2018 to 2024, during which the same respiratory virus target set was consistently available within a stable routine laboratory testing framework.

## 2. Materials and Methods

### 2.1. Study Design and Data Source

This retrospective observational study analyzed respiratory virus multiplex PCR data collected between January 2007 and December 2024 at a tertiary care hospital in the Republic of Korea. The 2007–2024 study period was selected because 2007 represented the earliest year when respiratory virus multiplex PCR results were continuously available in the institutional laboratory database, whereas 2024 was the most recent complete calendar year available at the time of data extraction and analysis.

This interval enabled comparison of long-term co-detection patterns across a pre-pandemic baseline period, the COVID-19 pandemic period, and the available post-pandemic period. Data were extracted from the laboratory database, including patient age and viral detection results for each testing episode. The unit of analysis was the individual testing episode rather than the patient. Repeated tests from the same patient were retained as separate testing episodes because the purpose of this study was to characterize episode-based laboratory surveillance patterns rather than patient-level infection incidence. Accordingly, the findings should be interpreted as reflecting temporal changes in episode-based co-detection patterns.

### 2.2. Study Period Classification

For temporal comparison, the study period was divided into three phases: pre-pandemic (2007–2019), pandemic (2020–2022), and post-pandemic (2023–2024). This classification was selected to reflect the period before the COVID-19 pandemic, that of major pandemic-related public health disruption, and the subsequent period of relaxation and reorganization of respiratory virus circulation.

### 2.3. Viral Targets and Definition of Co-Detection

The multiplex PCR panels included influenza A virus, influenza B virus, RSV A, RSV B, human metapneumovirus (hMPV), PIV types 1–3, rhinovirus, coronaviruses (229E, OC43, and NL63), adenovirus, enterovirus, and bocavirus, depending on the panel configuration each year.

Co-detection was defined as the detection of two or more respiratory viruses within the same testing episode, based on a single respiratory specimen analyzed by multiplex PCR. On this basis, each testing episode was classified as negative, single-virus detection, or co-detection. Because viral target composition varied across assay periods, temporal comparisons of co-detection burden and pairwise structure were interpreted within the tiered analytical framework described below.

### 2.4. Age Group Classification

Age was categorized into six groups: <1 year, 1–6, 7–12, 13–18, 19–64, and ≥65 years. Age-stratified analyses were performed to describe the relative distribution of co-detection episodes across age groups within each epidemiologic period. These analyses were intended to characterize the composition of observed co-detection episodes by age, rather than to estimate age-specific population risk.

### 2.5. Sensitivity Analysis for Panel Consistency

Because assay target composition varied across calendar periods, we adopted a tiered analytical framework to improve the validity and interpretability of temporal comparisons. First, analyses based on the full assay panel were treated as descriptive crude analyses, reflecting the overall observed co-detection burden within each assay period. Second, the primary temporal comparison was performed using a restricted analysis that included only 12 viral targets consistently available across the major study periods: influenza A virus, influenza B virus, RSV A, RSV B, hMPV, PIV types 1–3, rhinovirus, coronavirus 229E, coronavirus OC43, and adenovirus. This restricted framework was used to reduce bias arising from temporal changes in assay target composition. Third, a recent-period sensitivity analysis was conducted using data from 2018 to 2024, during which the same respiratory virus target set was consistently available within a stable routine laboratory testing framework. The recent-period sensitivity analysis included the viral targets consistently available from 2018 to 2024, including influenza A virus, influenza B virus, RSV A, RSV B, hMPV, PIV types 1–3, rhinovirus, coronaviruses 229E, OC43, and NL63, adenovirus, enterovirus, and bocavirus. This sensitivity analysis was intended to assess whether the temporal redistribution of dominant pairwise co-detection patterns persisted under a stable assay framework. Given methodological differences across the full study period, the present study focused primarily on relative temporal changes in co-detection burden and pairwise composition rather than on direct comparison of absolute detection frequencies across assay eras.

### 2.6. Pairwise Co-Detection Analysis

Viral co-detection patterns were summarized at the pairwise level. For episodes in which three or more viruses were detected, all possible two-virus combinations were generated and included as pairwise combinations of viruses.

Accordingly, the denominator for pairwise analysis was not the number of patients or co-detection episodes but the total number of pairwise combinations generated within each period; therefore, the results reflect combination-level composition rather than episode-level probability. The proportion of each viral pair represents its relative frequency among all pairwise combinations during that period. This exploratory analysis was designed to characterize shifts in the distribution and dominance of viral combinations, rather than to infer causal interactions between viruses. Because more than one pairwise combination could arise from a single testing episode when three or more viruses were detected, pairwise combinations were analyzed as descriptive combination-level units rather than as statistically independent patient-level observations.

### 2.7. Laboratory Testing

Respiratory virus testing was performed using multiplex PCR assays whose target composition varied across assay eras, with up to 15 viral targets included depending on the platform and calendar period. Nasopharyngeal swab specimens were collected and processed according to the laboratory’s internal standard operating procedures. Before analysis, samples were stored at 4 °C for no longer than 24 h. Viral nucleic acids were extracted using the QIAamp Viral RNA Mini Kit (QIAGEN, Hilden, Germany) in accordance with the manufacturer’s protocol. The assay panel was designed to detect both RNA and DNA respiratory viruses. All multiplex PCR tests were carried out in the hospital’s diagnostic laboratory as part of routine clinical practice, with quality maintained through established internal quality-control procedures and external quality-assessment programs.

From 2007 to 2012, respiratory viruses were detected using the Seeplex™ RV series multiplex PCR assay (Seegene, Seoul, Republic of Korea). During this period, PCR results were interpreted by gel electrophoresis following conventional endpoint PCR, and no additional sequencing was performed to confirm PCR amplicons. From 2013 onward, testing was transitioned to multiplex real-time PCR/RT-PCR assays according to the manufacturer’s instructions, using the AdvanSure™ RV and RV-Plus Real-Time RT-PCR kits and the SLAN Real-Time PCR System (LG Life Sciences, Seoul, Republic of Korea). Previous comparative evaluations have reported broadly comparable diagnostic performance between the Seeplex assay and the AdvanSure real-time RT-PCR assay [11,12]. Because the present study used long-term routine laboratory data across evolving diagnostic platforms, temporal interpretation emphasized harmonized restricted analyses and recent-period sensitivity analyses rather than direct platform-level performance comparisons.

### 2.8. Statistical Analysis

Categorical variables are presented as frequencies and percentages. Differences in the proportions of negative, single-virus detection, and co-detection episodes were evaluated using the chi-square test.

Temporal differences in the pairwise co-detection composition were assessed using contingency table analysis and chi-square tests. The same methods were applied in the sensitivity analysis restricted to the 2018–2024 period. 

Differences in the age-group distribution of co-detection episodes across periods were also evaluated using the chi-square test. Effect sizes for contingency table comparisons were assessed using Cramer’s V. Given the exploratory nature of the pairwise analyses and the presence of sparse categories, particularly in the recent-period sensitivity analysis, *p*-values were interpreted as indicators of global distributional heterogeneity rather than as confirmatory evidence for specific viral pairs. All statistical analyses were performed using the R software (R Foundation for Statistical Computing, version 4.3.3, Vienna, Austria). All tests were two-sided, and *p*-values of <0.05 were considered statistically significant.

### 2.9. Ethical Considerations

This study was conducted in accordance with the ethical principles of the Declaration of Helsinki (1975, revised in 2013) and STROBE reporting guidelines. The study protocol was approved by the Institutional Bioethics Committee of Dankook University (approval number: DKU 2025-02-004-003, Approval date: 23 April 2025). Because this retrospective study used de-identified data, the requirement for informed consent was waived.

## 3. Results

### 3.1. Overall Co-Detection Frequency

**Figure 1:**
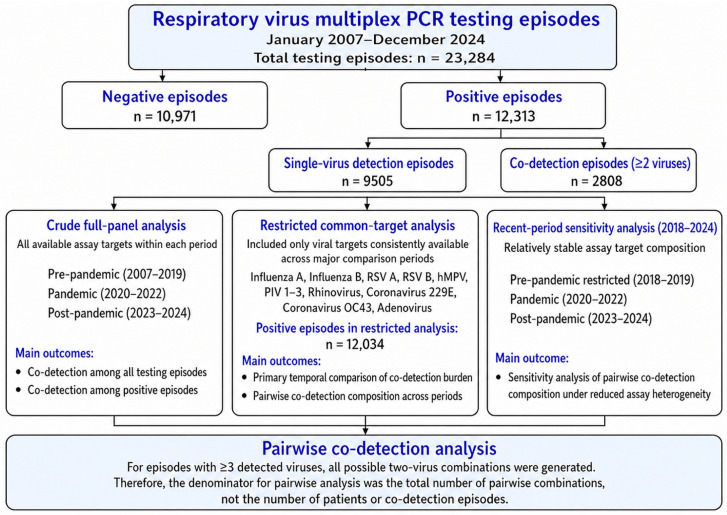
Analytical flow of respiratory virus multiplex PCR testing episodes and tiered analytic framework. The figure summarizes the classification of 23,284 respiratory virus multiplex PCR testing episodes from 2007 to 2024 and the three analytic tiers used to address assay heterogeneity: crude full-panel analysis, restricted common-target analysis, and recent-period sensitivity analysis. Pairwise co-detection analyses were based on all possible two-virus combinations generated from co-detection episodes. Single-virus detection episodes are included in the denominator when estimating co-detection burden among positive episodes in the crude full-panel, restricted common-target, and recent-period sensitivity analyses; however, pairwise co-detection composition analyses are restricted to co-detection episodes only.

As shown in Figure 1, a total of 23,284 respiratory virus multiplex PCR testing episodes were included in the analysis and were subsequently classified according to viral detection status and analytical framework. Among these, 12,313 (52.9%) episodes were positive for at least one virus. Single-virus detection was observed in 9505 (40.8%) episodes, whereas co-detection was observed in 2808 (12.1%) episodes. Among all positive episodes, co-detection accounted for 22.8% (Table 1).

### 3.2. Temporal Changes in Co-Detection Burden

**Figure 2:**
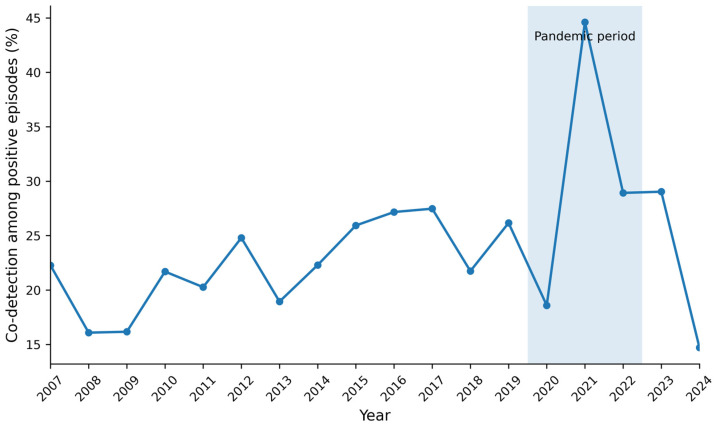
Annual trends in co-detection burden in the crude full-panel analysis. The blue line connects the annual observed proportions of co-detection among positive respiratory virus PCR testing episodes.

In this section, co-detection refers to the detection of two or more respiratory viruses within a single testing episode by multiplex PCR. Temporal trends in co-detection burden differed according to the analytical framework used. In the crude analysis based on the full assay panel, the proportion of co-detection among positive episodes was 22.4% in the pre-pandemic period, 30.1% during the pandemic, and 24.3% in the post-pandemic period (Table 1). Annual crude trends are shown in Figure 2. The apparent increase observed in the crude full-panel analysis should be interpreted cautiously, because temporal comparisons across assay periods are vulnerable to bias introduced by changes in target composition over time.

By contrast, in the restricted common-target analysis used for the primary temporal comparison, the proportion of co-detection among positive episodes decreased stepwise from 20.5% before the pandemic to 14.8% during the pandemic and 12.2% after the pandemic, indicating that the apparent increase observed in the crude full-panel analysis was not robust to target harmonization (Table S1). The corresponding annual trends are presented in Figure S1.

The figure shows the annual proportion of co-detection among positive respiratory virus PCR testing episodes from 2007 to 2024. The shaded area indicates the pandemic period (2020–2022). Because assay target composition changed over time, these crude trends should be interpreted together with the restricted common-target analysis shown in Figure S1.

### 3.3. Restructuring of Dominant Pairwise Co-Detection Patterns

**Figure 3:**
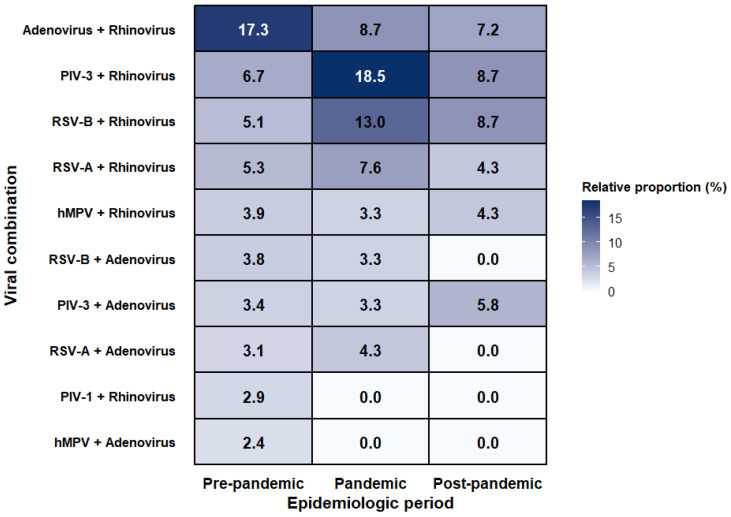
Heatmap of the redistribution of dominant pairwise respiratory viral co-detection patterns across epidemiologic periods in the restricted common-target analysis.

In the restricted analysis, the composition of pairwise co-detection differed across periods (χ^2^ = 58.14, df = 20, *p* < 0.001; Cramer’s V = 0.090), indicating statistically significant but modest global compositional heterogeneity (Table 2). Before the pandemic, adenovirus–rhinovirus accounted for the largest relative share among pairwise co-detection combinations (17.3%), followed by PIV type 3–rhinovirus (6.7%), RSV A–rhinovirus (5.3%), and RSV B–rhinovirus (5.1%). During the pandemic, rhinovirus-centered combinations involving PIV type 3 (18.5%) or RSV B (13.0%) accounted for larger relative shares within the observed pairwise co-detection distribution, whereas the proportion of adenovirus–rhinovirus decreased to 8.7%. In the post-pandemic period, adenovirus–rhinovirus remained at a comparably reduced share (7.2%), while PIV type 3–rhinovirus and RSV B–rhinovirus each accounted for 8.7% of pairwise co-detection combinations. These shifts in pairwise composition are illustrated in Figure 3.

The heatmap displays the relative proportion (%) of the major pairwise respiratory viral co-detection combinations across three epidemiologic periods: pre-pandemic, pandemic, and post-pandemic. Values in each cell indicate the proportion of each viral pair among all pairwise co-detection combinations observed within that period. In episodes with three or more detected viruses, all possible two-virus combinations are generated and included in the analysis. The figure highlights temporal restructuring of pairwise composition across periods and should not be interpreted as reflecting episode-level co-detection burden or direct biological interaction between viruses. Abbreviation: hMPV, human metapneumovirus. Relative proportion (%) is calculated as the number of the indicated pairwise viral combination divided by the total number of pairwise co-detection combinations observed within the same epidemiologic period × 100.

### 3.4. Sensitivity Analysis Using Recent Data

In the sensitivity analysis restricted to the recent period (2018–2024), during which the same respiratory virus target set was consistently available within a stable routine laboratory testing framework, pairwise co-detection patterns also showed exploratory evidence of distributional heterogeneity across periods (χ^2^ = 244.73, df = 160, *p* < 0.001; Cramer’s V = 0.366; Table S2). Rhinovirus-containing pairs remained central throughout all periods, although their relative contributions varied over time. Notably, the rhinovirus–enterovirus combination accounted for 15.0% in the pre-pandemic period (2018–2019), 33.6% during the pandemic, and 25.6% in the post-pandemic period. These patterns are illustrated in Figure S2.

### 3.5. Age-Stratified Distribution

The age distribution of co-detection episodes differed across periods (χ^2^ = 240.33, df = 10, *p* < 0.001; Cramer’s V = 0.207) (Table 3).

In all periods, most co-detection episodes occurred in children aged <7 years. Although the relative proportion of adults and older adults was greater in the post-pandemic period than in earlier periods, this finding should be interpreted cautiously because the analysis describes the distribution of observed co-detection episodes rather than age-specific population risk and because the absolute number of non-pediatric co-detection episodes was small.

## 4. Discussion

This 18-year single-center analysis yielded three principal findings. First, temporal trends in co-detection burden were not robust to assay panel changes: the apparent pandemic-period increase in the crude full-panel analysis contrasted with a stepwise decrease in the target-restricted analysis. Second, the redistribution of dominant pairwise composition was more consistently observed across analytical frameworks, with adenovirus–rhinovirus predominance before the pandemic shifting toward rhinovirus-centered pairings involving PIV type 3 or RSV B during and after the pandemic. Third, the recent-period sensitivity analysis, based on a stable target set, further supported pairwise restructuring, with rhinovirus–enterovirus emerging as a leading pair. Together, these findings indicate that an important post-pandemic signal in respiratory virus surveillance may lie less in how often co-detection occurs than in which viral pairs predominate. This means that aggregate co-detection frequency alone may not fully reflect post-pandemic changes in respiratory virus circulation, whereas shifts in dominant pairwise composition may provide additional information on altered temporal overlap among circulating respiratory viruses.

These findings are broadly consistent with surveillance reports from Japan and the United States indicating that the COVID-19 pandemic substantially impacted the circulation and seasonality of multiple respiratory viruses, with post-pandemic recovery occurring asynchronously rather than uniformly [13,14]. Our results extend this literature by suggesting that post-pandemic respiratory viral reorganization may also be reflected in the structure of dominant co-detected viral pairs.

An important methodological implication of this study is that the interpretation of temporal changes in co-detection burden depended strongly on how assay heterogeneity was managed [15,16]. In the crude full-panel analysis, co-detection among positive episodes appeared to increase during the pandemic. In contrast, in the restricted analysis based on common viral targets, the same proportion decreased progressively across pre-pandemic, pandemic, and post-pandemic periods. This discrepancy indicates that long-term multiplex PCR datasets may reflect not only epidemiologic change but also shifts in assay target composition and platform structure [17]. Accordingly, comparisons of co-detection burden over extended periods should be interpreted cautiously, and harmonized target-restricted analyses may provide a more robust basis for temporal inference.

By contrast, the redistribution of pairwise co-detection patterns appeared to be a relatively consistent descriptive signal across the analytical frameworks. In the restricted 12-target analysis, adenovirus–rhinovirus predominated before the pandemic, whereas rhinovirus-containing pairs involving parainfluenza virus type 3 or RSV B accounted for a larger share during and after the pandemic. Quantitatively, this redistribution was reflected by a decline in adenovirus–rhinovirus from 17.3% before the pandemic to 8.7% during and 7.2% after the pandemic, alongside increased relative shares of PIV type 3–rhinovirus and RSV B–rhinovirus during the pandemic [18,19,20]. Rather than indicating a simple shift in overall detection frequency, these findings may reflect changes in temporal overlap among circulating viruses, differences in the tested population, and the composition of multiplex assay capture. The repeated prominence of rhinovirus-containing pairs is also notable. Previous studies have suggested that rhinovirus circulation showed relatively strong persistence during periods of non-pharmaceutical intervention and often re-emerged earlier than several enveloped respiratory viruses [21,22,23,24]. In this context, the present findings may reflect sustained rhinovirus circulation and repeated overlap with other viruses. Although mechanisms such as viral interference have been proposed [25,26], the present study was not designed to test such mechanisms directly; therefore, the observed pairwise redistribution should be interpreted primarily as a descriptive surveillance pattern rather than evidence of direct biological interaction.

The recent-period sensitivity analysis provided additional support for the temporal redistribution of pairwise co-detection composition. When the analysis was restricted to 2018–2024, during which the same respiratory virus target set was consistently available within a stable routine laboratory testing framework, pairwise co-detection patterns showed exploratory evidence of distributional heterogeneity across periods, and rhinovirus–enterovirus emerged as the most prominent pair in this framework. This finding supports the interpretation that the redistribution of pairwise composition was not attributable solely to long-term changes in assay target composition. However, the difference in the leading pair between the restricted 12-target analysis and the recent-period sensitivity analysis indicates that the exact rank order of pairwise combinations may vary depending on the analytical framework and included viral targets. Age distributions also differed across periods. Co-detection remained concentrated in young children throughout the study period, consistent with previous reports that multiple-virus detections are most common in pediatric populations [27,28,29,30]. The relative share of adults and older adults among co-detection episodes was greater in the post-pandemic period; however, because the absolute number of non-pediatric co-detection episodes was small, this pattern should not be interpreted as evidence of increased age-specific risk. Rather, it is better understood as a distributional feature within the observed co-detection dataset.

From a practical perspective, these findings may have implications beyond laboratory diagnosis. In the post-pandemic era, monitoring dominant co-detected viral pairs may help clinicians interpret multiplex PCR results within a broader epidemiologic context, particularly when respiratory virus circulation deviates from historical seasonal patterns. Although the present study does not establish that specific viral pairs are associated with greater disease severity or require different patient-level management, recognizing shifts in co-detection composition may support more contextual interpretation of respiratory virus surveillance data and inform hospital-level preparedness during periods of atypical viral circulation. Further studies linking pairwise co-detection patterns with clinical severity, treatment, and outcome data are needed to clarify their direct clinical relevance.

A major strength of this study is the inclusion of an 18-year laboratory surveillance dataset spanning the pre-pandemic, pandemic, and post-pandemic periods. Additional strengths include the focus on pairwise co-detection composition rather than only overall positivity or co-detection burden, and the use of both a restricted common-target framework and a recent-period sensitivity analysis to improve temporal interpretability despite changes in assay target composition over time.

Some limitations should be acknowledged. First, this was a retrospective single-center study, and the findings may not be generalizable to other institutions or community settings. Second, the unit of analysis was the testing episode rather than the individual patient; accordingly, repeated testing of the same patient may have influenced the observed distributions and should not be interpreted as patient-level incidence. Third, pairwise counts were derived from all possible two-virus combinations within co-detection episodes, meaning that the pairwise analysis reflects combination-level composition rather than episode-level probability. Fourth, clinical data on disease severity, symptoms, treatment, and outcomes were unavailable, precluding assessment of the clinical significance of specific co-detection pairs. Fifth, multiplex PCR detection of multiple viral nucleic acids in a single specimen does not necessarily reflect simultaneous active co-replication. Such results may also arise from sequential infections with residual nucleic acid shedding or asymptomatic carriage. Therefore, all pairwise patterns reported in this study should be interpreted as laboratory co-detection rather than confirmed viral co-infection. Finally, residual heterogeneity related to assay platform and target composition cannot be fully excluded across the full 2007–2024 study period, despite using restricted and recent-period sensitivity analyses. The pairwise analysis should therefore be interpreted as exploratory and descriptive rather than as evidence of causal or synergistic viral interactions.

Overall, these findings suggest that interpretation of post-pandemic respiratory virus surveillance may be strengthened by moving beyond single-virus positivity to include the composition and temporal redistribution of co-detected viral pairs. Further multicenter studies using standardized assay panels and linked clinical data are needed to determine whether these temporal pairwise patterns are reproducible across settings and to clarify the extent to which they reflect changing viral overlap, differences in tested populations, or broader ecological reorganization. In long-term respiratory virus surveillance, an important post-pandemic impact may be reflected not only in how often co-detection occurs, but also in how dominant pairwise structures are reorganized over time.

## 5. Conclusions

In this long-term single-center analysis, the most consistent post-pandemic impact on respiratory viral co-detection was not a uniform change in overall co-detection burden, but a temporal redistribution of pairwise co-detection composition. Although the direction of change in co-detection burden varied by analytical framework, changes in pairwise composition appeared more stable across analyses. Rhinovirus-containing pairs remained central, while the relative prominence of specific viral combinations shifted across periods. These findings support cautious interpretation of long-term co-detection trends derived from multiplex PCR datasets collected across changing assay platforms. These results highlight the value of incorporating pairwise co-detection structure into post-pandemic respiratory virus surveillance, alongside conventional single-virus positivity and overall co-detection frequency.

## Figures and Tables

**Table 1:** Distribution of respiratory virus PCR testing episodes by detection status across study periods.

Category	Pre-Pandemic (2007–2019)	Pandemic (2020–2022)	Post-Pandemic (2023–2024)	Total
Total testing episodes	19,350	2265	1669	23,284
Negative episodes	7951	1766	1254	10,971
Positive episodes	11,399	499	415	12,313
Single-virus detection	8842	349	314	9505
Co-detection episodes	2557	150	101	2808
Co-detection among all episodes (%)	13.2	6.6	6.1	12.1
Co-detection among positive episodes (%)	22.4	30.1	24.3	22.8

Note: Values are presented as counts or percentages. Co-detection is defined as the simultaneous detection of two or more respiratory viruses in a single specimen. The unit of analysis is the testing episode. If *a* denotes co-detection episodes, *b* denotes single-virus detection episodes, and *c* denotes negative episodes within each period, co-detection among positive episodes is calculated as *a*/(*a* + *b*) × 100, whereas co-detection among all episodes is calculated as *a*/(*a* + *b* + *c*) × 100. Positive episodes are defined as *a* + *b*. This table reflects the crude full-panel analysis and should be interpreted alongside the restricted common-target analysis shown in Table S1.

**Table 2:** Major pairwise respiratory viral co-detection patterns across study periods in the restricted analysis.

Viral Combination	Pre-Pandemic *n* (%)	Pandemic *n* (%)	Post-Pandemic *n* (%)	Total *n* (%)
Adenovirus + Rhinovirus	588 (17.3)	8 (8.7)	5 (7.2)	601 (16.9)
PIV type 3 + Rhinovirus	228 (6.7)	17 (18.5)	6 (8.7)	251 (7.1)
RSV B + Rhinovirus	174 (5.1)	12 (13.0)	6 (8.7)	192 (5.4)
RSV A + Rhinovirus	179 (5.3)	7 (7.6)	3 (4.3)	189 (5.3)
hMPV + Rhinovirus	132 (3.9)	3 (3.3)	3 (4.3)	138 (3.9)
RSV B + Adenovirus	128 (3.8)	3 (3.3)	0 (0.0)	131 (3.7)
PIV type 3 + Adenovirus	116 (3.4)	3 (3.3)	4 (5.8)	123 (3.5)
RSV A + Adenovirus	104 (3.1)	4 (4.3)	0 (0.0)	108 (3.0)
PIV type 1 + Rhinovirus	97 (2.9)	0 (0.0)	0 (0.0)	97 (2.7)
hMPV + Adenovirus	83 (2.4)	0 (0.0)	0 (0.0)	83 (2.3)
Others	1569 (46.2)	35 (38.0)	42 (60.9)	1646 (46.2)
Total	3398 (100.0)	92 (100.0)	69 (100.0)	3559 (100.0)

Note: This table is based on the restricted analysis including 12 viral targets consistently available across the major study periods. Percentages represent the proportion of each viral pair among all pairwise co-detection combinations within each period. For episodes with three or more detected viruses, all possible two-virus combinations were generated; therefore, pairwise observations are not considered independent patient-level events. The 10 pairwise combinations with the highest overall frequencies in the restricted analysis are shown individually, while the remaining lower-frequency pairwise viral combinations are grouped as “Others.” Accordingly, this table summarizes the dominant pairwise structure, and chi-square results were interpreted descriptively as evidence of global compositional heterogeneity rather than as confirmatory tests of individual pairwise differences. PIV, parainfluenza virus; RSV, respiratory syncytial virus.

**Table 3:** Age distribution of co-detection episodes across study periods.

Age Group (Years)	Pre-Pandemic *n* (%)	Pandemic *n* (%)	Post-Pandemic *n* (%)	Total *n* (%)
<1	822 (32.1)	48 (32.0)	12 (11.9)	882 (31.4)
1–6	1514 (59.2)	84 (56.0)	44 (43.6)	1642 (58.5)
7–12	86 (3.4)	6 (4.0)	2 (2.0)	94 (3.3)
13–18	15 (0.6)	1 (0.7)	1 (1.0)	17 (0.6)
19–64	50 (2.0)	3 (2.0)	13 (12.9)	66 (2.4)
≥65	70 (2.7)	8 (5.3)	29 (28.7)	107 (3.8)

Note: Percentages represent the proportion of each age group among all co-detection episodes within each period. Interpretation of age-specific patterns in the post-pandemic period should be made with caution, owing to the relatively small number of non-pediatric co-detection episodes.

## Data Availability

The data presented in this study are available on reasonable request from the corresponding author due to privacy, ethical, and institutional data-sharing restrictions.

## Supplementary Materials

The following supporting information can be downloaded at: https://www.mdpi.com/article/10.3390/microorganisms14051134/s1, Figure S1: Annual co-detection burden in the restricted analysis; Figure S2: Temporal redistribution of major pairwise respiratory viral co-detection patterns in the recent-period sensitivity analysis (2018–2024); Table S1: Restricted analysis of respiratory viral co-detection burden using viral targets consistently available across epidemiologic periods; Table S2: Pairwise respiratory viral co-detection patterns in the recent-period sensitivity analysis using a stable respiratory virus target set (2018–2024).

## Abbreviations

The following abbreviations are used in this manuscript:

COVID-19	Coronavirus disease
PIV	Parainfluenza virus
RSV	Respiratory syncytial virus
