# Supplementary material for: Pairwise Respiratory Viral Co-Detection Patterns Before, During, and After the COVID-19 Pandemic: An 18-Year Multiplex PCR Surveillance Study
Source: Microorganisms. 2026 May 16;14(5):1134. doi: 10.3390/microorganisms14051134 (PMC13209395; doi:10.3390/microorganisms14051134)
Supplement: Supplementary file 1 [file microorganisms-14-01134-s001.zip › microorganisms-4317335-supplementary.pdf]

Table S1. Restricted analysis of respiratory viral co-detection burden using viral targets consistently available acr oss epidemiologic periods

Variable	Pre-pandemic (2007–2019)	Pandemic (2020–2022)	Post-pandemic (2023–2024)	Total, n
Positive episodes in restricted common-target analysis	11,182	460	392	12,034
Single-virus detection episodes	8894	392	344	9630
Co-detection episodes	2288	68	48	2404
Co-detection among positive episodes, %	20.5	14.8	12.2	20.0

Notes: This restricted analysis includes only viral targets consistently available across the major comparison perio ds: influenza A, influenza B, RSV A, RSV B, human metapneumovirus, parainfluenza virus types 1–3, rhinovirus, coronavirus 229E, coronavirus OC43, and adenovirus. Viral co-detection was defined as simultaneous detection o f two or more viruses in the same specimen. This analysis is performed to reduce bias related to changes in assay target composition over time.

Table S2. Pairwise respiratory viral co-detection patterns in the recent-period sensitivity analysis using a stable re spiratory virus target set (2018–2024)

Viral pair	Pre-pandemic restricted (2018–2019), n (%)	Pandemic (2020–2022), n (%)	Post-pandemic (2023–2024), n (%)	Total, n (%)
Rhinovirus + Enterovirus	80 (15.0)	74 (33.6)	41 (25.6)	195 (21.4)
Rhinovirus + Adenovirus	66 (12.4)	8 (3.6)	5 (3.1)	79 (8.7)
Rhinovirus + Bocavirus	35 (6.6)	15 (6.8)	6 (3.8)	56 (6.1)
PIV type 3 + Rhinovirus	20 (3.8)	17 (7.7)	6 (3.8)	43 (4.7)
Adenovirus + Bocavirus	25 (4.7)	8 (3.6)	3 (1.9)	36 (3.9)
Adenovirus + Enterovirus	25 (4.7)	3 (1.4)	7 (4.4)	35 (3.8)
RSV B + Rhinovirus	11 (2.1)	12 (5.5)	6 (3.8)	29 (3.2)
PIV type 3 + Bocavirus	16 (3.0)	6 (2.7)	5 (3.1)	27 (3.0)
Enterovirus + Bocavirus	17 (3.2)	4 (1.8)	3 (1.9)	24 (2.6)
RSV A + Rhinovirus	14 (2.6)	7 (3.2)	3 (1.9)	24 (2.6)
hMPV + Rhinovirus	13 (2.4)	3 (1.4)	3 (1.9)	19 (2.1)
PIV type 3 + Adenovirus	12 (2.3)	3 (1.4)	4 (2.5)	19 (2.1)
PIV type 3 + Enterovirus	3 (0.6)	8 (3.6)	6 (3.8)	17 (1.9)
Coronavirus OC43 + Adenovirus	7 (1.3)	4 (1.8)	5 (3.1)	16 (1.8)
RSV A + Coronavirus OC43	12 (2.3)	1 (0.5)	3 (1.9)	16 (1.8)
RSV A + Adenovirus	11 (2.1)	4 (1.8)	0 (0.0)	15 (1.6)
hMPV + Adenovirus	10 (1.9)	1 (0.5)	1 (0.6)	12 (1.3)
RSV A + Bocavirus	6 (1.1)	5 (2.3)	1 (0.6)	12 (1.3)
Influenza A + Adenovirus	9 (1.7)	0 (0.0)	1 (0.6)	10 (1.1)
Influenza A + RSV A	9 (1.7)	1 (0.5)	0 (0.0)	10 (1.1)
Rhinovirus + Coronavirus OC43	6 (1.1)	1 (0.5)	2 (1.2)	9 (1.0)
Influenza A + Coronavirus 229E	5 (0.9)	1 (0.5)	2 (1.2)	8 (0.9)
hMPV + Enterovirus	5 (0.9)	1 (0.5)	2 (1.2)	8 (0.9)
Coronavirus NL63 + Adenovirus	7 (1.3)	0 (0.0)	1 (0.6)	8 (0.9)
PIV type 1 + Rhinovirus	5 (0.9)	2 (0.9)	1 (0.6)	8 (0.9)
Influenza A + RSV B	2 (0.4)	1 (0.5)	4 (2.5)	7 (0.8)
RSV A + Enterovirus	4 (0.8)	0 (0.0)	3 (1.9)	7 (0.8)
PIV type 1 + Bocavirus	5 (0.9)	0 (0.0)	2 (1.2)	7 (0.8)
PIV type 3 + Coronavirus OC43	2 (0.4)	2 (0.9)	3 (1.9)	7 (0.8)
Influenza A + Influenza B	6 (1.1)	0 (0.0)	0 (0.0)	6 (0.7)
Influenza A + Coronavirus NL63	3 (0.6)	1 (0.5)	2 (1.2)	6 (0.7)
Influenza A + Coronavirus OC43	3 (0.6)	2 (0.9)	1 (0.6)	6 (0.7)
hMPV + Bocavirus	5 (0.9)	0 (0.0)	1 (0.6)	6 (0.7)
hMPV + PIV type 3	3 (0.6)	0 (0.0)	3 (1.9)	6 (0.7)
Coronavirus OC43 + Bocavirus	2 (0.4)	2 (0.9)	2 (1.2)	6 (0.7)

Viral pair	Pre-pandemic restricted (2018–2019), n (%)	Pandemic (2020–2022), n (%)	Post-pandemic (2023–2024), n (%)	Total, n (%)
RSV B + Adenovirus	3 (0.6)	3 (1.4)	0 (0.0)	6 (0.7)
Influenza A + Rhinovirus	2 (0.4)	2 (0.9)	1 (0.6)	5 (0.5)
RSV A + PIV type 3	2 (0.4)	2 (0.9)	1 (0.6)	5 (0.5)
RSV B + Enterovirus	0 (0.0)	2 (0.9)	3 (1.9)	5 (0.5)
RSV B + PIV type 3	1 (0.2)	2 (0.9)	2 (1.2)	5 (0.5)
Rhinovirus + Coronavirus NL63	5 (0.9)	0 (0.0)	0 (0.0)	5 (0.5)
Influenza B + Coronavirus OC43	3 (0.6)	0 (0.0)	1 (0.6)	4 (0.4)
PIV type 2 + Rhinovirus	4 (0.8)	0 (0.0)	0 (0.0)	4 (0.4)
Coronavirus 229E + Adenovirus	3 (0.6)	0 (0.0)	0 (0.0)	3 (0.3)
Coronavirus 229E + Coronavirus NL63	2 (0.4)	0 (0.0)	1 (0.6)	3 (0.3)
Influenza A + Bocavirus	2 (0.4)	0 (0.0)	1 (0.6)	3 (0.3)
Influenza B + Adenovirus	3 (0.6)	0 (0.0)	0 (0.0)	3 (0.3)
Influenza B + Coronavirus 229E	3 (0.6)	0 (0.0)	0 (0.0)	3 (0.3)
Influenza B + RSV B	3 (0.6)	0 (0.0)	0 (0.0)	3 (0.3)
Coronavirus OC43 + Enterovirus	0 (0.0)	2 (0.9)	1 (0.6)	3 (0.3)
Coronavirus OC43 + Coronavirus NL63	2 (0.4)	1 (0.5)	0 (0.0)	3 (0.3)
RSV A + PIV type 2	0 (0.0)	3 (1.4)	0 (0.0)	3 (0.3)
RSV B + Coronavirus 229E	2 (0.4)	1 (0.5)	0 (0.0)	3 (0.3)
RSV B + Human metapneumovirus	2 (0.4)	0 (0.0)	1 (0.6)	3 (0.3)
RSV B + Coronavirus OC43	2 (0.4)	1 (0.5)	0 (0.0)	3 (0.3)
PIV type 1 + Adenovirus	2 (0.4)	0 (0.0)	1 (0.6)	3 (0.3)
PIV type 3 + Coronavirus NL63	2 (0.4)	0 (0.0)	1 (0.6)	3 (0.3)
hMPV + Coronavirus NL63	2 (0.4)	0 (0.0)	0 (0.0)	2 (0.2)
hMPV + Coronavirus OC43	1 (0.2)	0 (0.0)	1 (0.6)	2 (0.2)
hMPV + PIV type 1	2 (0.4)	0 (0.0)	0 (0.0)	2 (0.2)
Coronavirus NL63 + Bocavirus	2 (0.4)	0 (0.0)	0 (0.0)	2 (0.2)
RSV A + hMPV	1 (0.2)	1 (0.5)	0 (0.0)	2 (0.2)
RSV A + PIV type 1	1 (0.2)	0 (0.0)	1 (0.6)	2 (0.2)
PIV type 1 + Enterovirus	0 (0.0)	1 (0.5)	1 (0.6)	2 (0.2)
PIV type 1 + Coronavirus OC43	0 (0.0)	0 (0.0)	2 (1.2)	2 (0.2)
PIV type 1 + PIV type 3	1 (0.2)	0 (0.0)	1 (0.6)	2 (0.2)
Coronavirus 229E + Bocavirus	1 (0.2)	0 (0.0)	0 (0.0)	1 (0.1)
Influenza A + Enterovirus	0 (0.0)	0 (0.0)	1 (0.6)	1 (0.1)
Influenza A + hMPV	1 (0.2)	0 (0.0)	0 (0.0)	1 (0.1)
Influenza A + PIV type 1	1 (0.2)	0 (0.0)	0 (0.0)	1 (0.1)
Influenza A + PIV type 2	1 (0.2)	0 (0.0)	0 (0.0)	1 (0.1)

Viral pair	Pre-pandemic restricted (2018–2019), n (%)	Pandemic (2020–2022), n (%)	Post-pandemic (2023–2024), n (%)	Total, n (%)
Influenza A + PIV type 3	0 (0.0)	0 (0.0)	1 (0.6)	1 (0.1)
Influenza B + Bocavirus	1 (0.2)	0 (0.0)	0 (0.0)	1 (0.1)
Influenza B + hMPV	1 (0.2)	0 (0.0)	0 (0.0)	1 (0.1)
Influenza B + Coronavirus NL63	1 (0.2)	0 (0.0)	0 (0.0)	1 (0.1)
hMPV + PIV type 2	1 (0.2)	0 (0.0)	0 (0.0)	1 (0.1)
RSV A + Coronavirus 229E	0 (0.0)	1 (0.5)	0 (0.0)	1 (0.1)
RSV A + Coronavirus NL63	1 (0.2)	0 (0.0)	0 (0.0)	1 (0.1)
RSV A + RSV B	0 (0.0)	1 (0.5)	0 (0.0)	1 (0.1)
PIV type 2 + Adenovirus	1 (0.2)	0 (0.0)	0 (0.0)	1 (0.1)
PIV type 2 + Enterovirus	1 (0.2)	0 (0.0)	0 (0.0)	1 (0.1)
Total pairwise combinations	533 (100.0)	220 (100.0)	160 (100.0)	913 (100.0)

Notes: This sensitivity analysis is restricted to the recent period (2018–2024), during which the same respiratory virus target set is consistently available within a stable routine laboratory testing framework, to confirm whether temporal redistribution of dominant pairwise co-detections persisted under a stable target framework. Percentages indicate the proportion of each viral pair among all pairwise co-detection occurrences within the corresponding epidemiologic period.

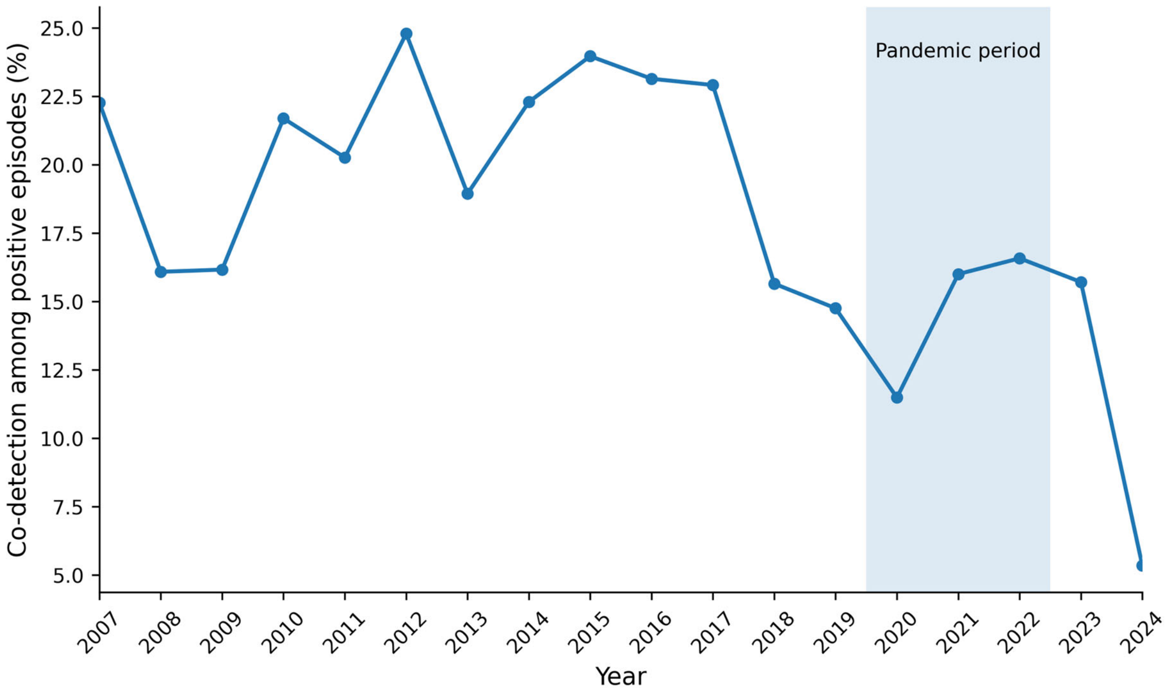

Figure S1. Annual co-detection burden in the restricted analysis. Annual co-detection proportions among positiv e respiratory virus PCR testing episodes based on the restricted analysis, including only viral targets consistently available across the major comparison periods.

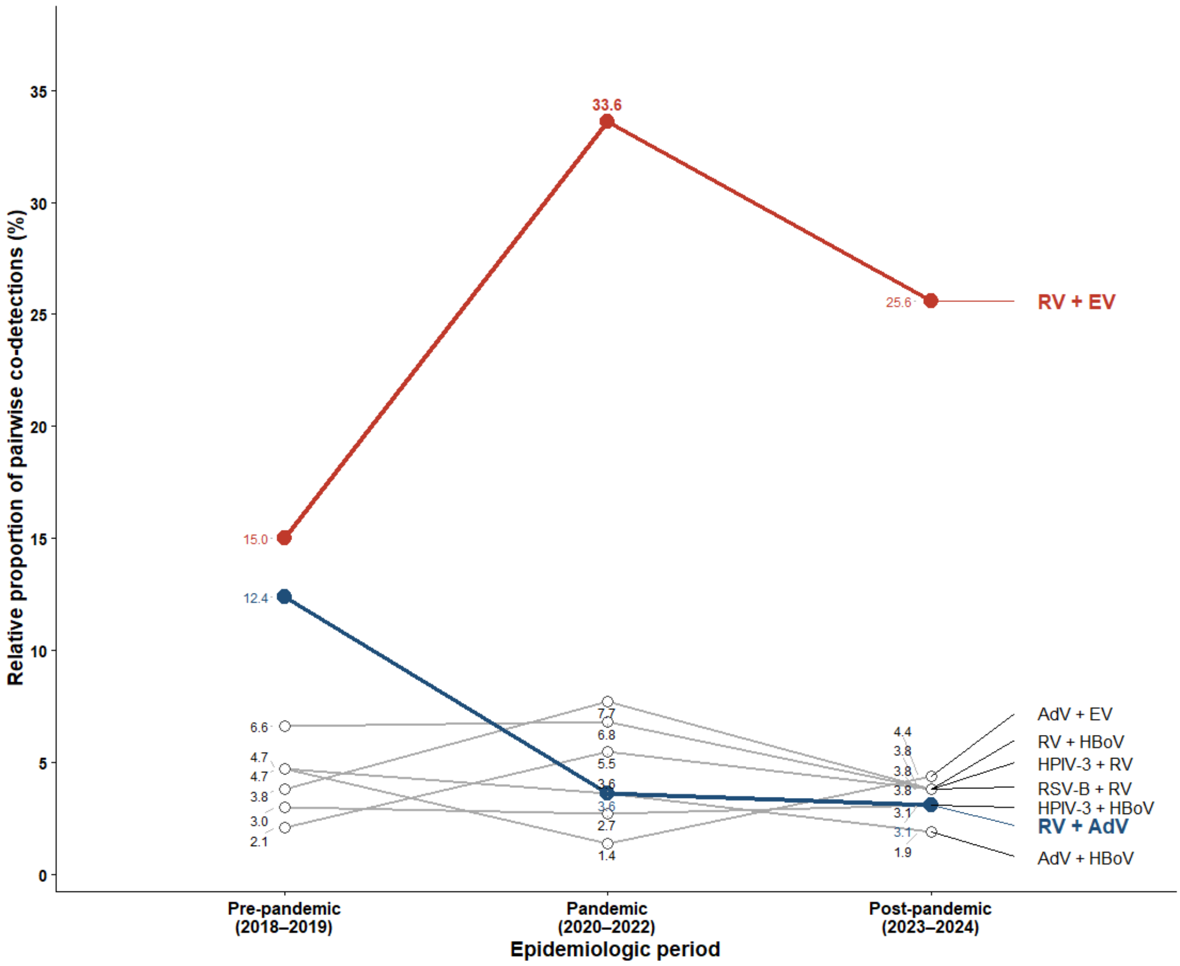

Figure S2. Temporal redistribution of major pairwise respiratory viral co-detection patterns in the recent-period sensitivity analysis (2018–2024).

Connected dot plot showing the relative proportion (%) of the eight major pairwise respiratory viral co-detection combinations in the recent-period sensitivity analysis restricted to 2018–2024, during which the same respiratory virus target set is consistently available within a stable routine laboratory testing framework. Each line represents one viral pair, and each point indicates its relative proportion among all pairwise co-detection occurrences within the corresponding epidemiologic period. The highlighted combinations illustrate the marked expansion of rhinovirus + enterovirus and the contraction of rhinovirus + adenovirus across periods.
